# BARM and BalticMicrobeDB, a reference metagenome and interface to meta-omic data for the Baltic Sea

**DOI:** 10.1038/sdata.2018.146

**Published:** 2018-07-31

**Authors:** Johannes Alneberg, John Sundh, Christin Bennke, Sara Beier, Daniel Lundin, Luisa W. Hugerth, Jarone Pinhassi, Veljo Kisand, Lasse Riemann, Klaus Jürgens, Matthias Labrenz, Anders F. Andersson

**Affiliations:** 1 KTH Royal Institute of Technology, Science for Life Laboratory, Department of Gene Technology, School of Engineering Sciences in Chemistry, Biotechnology and Health, 17165 Solna, Sweden; 2 Science for Life Laboratory, Department of Biochemistry and Biophysics, Stockholm University, 17165 Solna, Sweden; 3 Leibniz Institute for Baltic Sea Research Warnemünde, 18119 Rostock, Germany; 4 Centre for Ecology and Evolution in Microbial Model Systems, Linnaeus University, 39182 Kalmar, Sweden; 5 Centre for Translational Microbiome Research, Department of Molecular, Tumor and Cell Biology, Karolinska Institutet, Science for Life Laboratory, 17165 Solna, Sweden; 6 University of Tartu, Institute of Technology, 50411 Tartu, Estonia; 7 Section for Marine Biological Section, Department of Biology, University of Copenhagen, 3000 Helsingør, Denmark

**Keywords:** Genome informatics, Environmental sciences, Water microbiology, DNA sequencing, Metagenomics

## Abstract

The Baltic Sea is one of the world’s largest brackish water bodies and is characterised by pronounced physicochemical gradients where microbes are the main biogeochemical catalysts. Meta-omic methods provide rich information on the composition of, and activities within, microbial ecosystems, but are computationally heavy to perform. We here present the Baltic Sea Reference Metagenome (BARM), complete with annotated genes to facilitate further studies with much less computational effort. The assembly is constructed using 2.6 billion metagenomic reads from 81 water samples, spanning both spatial and temporal dimensions, and contains 6.8 million genes that have been annotated for function and taxonomy. The assembly is useful as a reference, facilitating taxonomic and functional annotation of additional samples by simply mapping their reads against the assembly. This capability is demonstrated by the successful mapping and annotation of 24 external samples. In addition, we present a public web interface, BalticMicrobeDB, for interactive exploratory analysis of the dataset.

## Background & Summary

**Figure 1:**
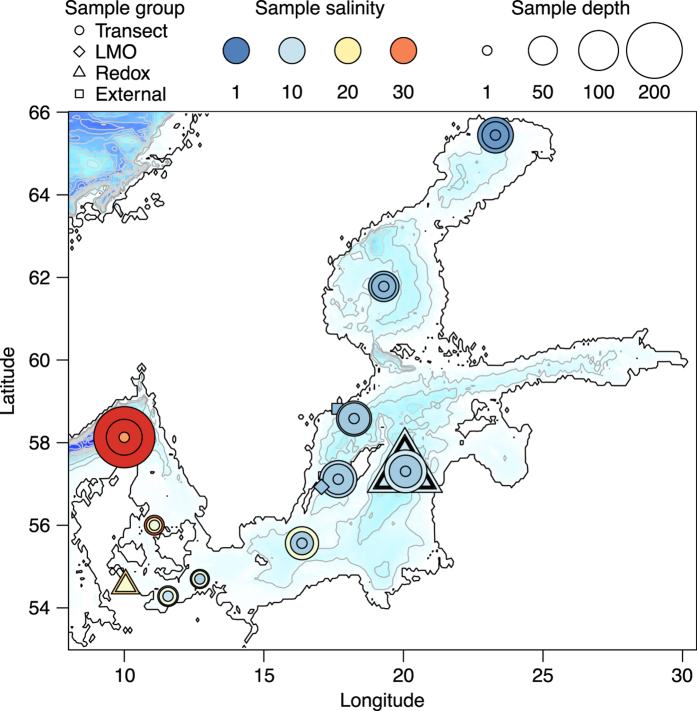
A map showing the locations for all stations where samples were taken. The three sample groups included in the assembly (Transect, LMO and Redoxcline) are displayed together with the external sample set^20^ (External), all groups indicated with different markers. The colour of the marker indicates the salinity of the water sample while the size indicates the depth at which it was taken. The background color indicates depth (from white to dark blue), with contour lines drawn with 50 m intervals. The map was generated using the Marmap package^36^ in *R*^37^ with bathymetric data from the ETOPO1 dataset hosted on the NOAA server^38^.

The Baltic Sea is a semi-enclosed inland sea characterized by strong physicochemical gradients, in particular horizontal and vertical salinity and oxygen gradients, and pronounced seasonal dynamics^1^. This ecosystem is also heavily impacted by anthropogenic eutrophication, manifested in e.g. harmful phytoplankton blooms and large areas with anoxic bottom waters^2^. Due to their key roles in biogeochemical cycles, microbial communities are particularly interesting to study in this ecosystem^3–11^. One of the most comprehensive methods to characterize the taxonomic and functional composition of microbial communities is through metagenomics, and specifically by metagenomic assembly, which enables high precision and sensitivity for both taxonomic and functional annotation^12^. These annotations can be quantified in individual samples by mapping short reads from samples that either were included in the assembly or constitute external samples. For some microbiomes, particularly those associated with the human body, extensive sequencing efforts have been undertaken to construct reference gene catalogues that are publicly available and can be utilized by others^13–15^. Large-scale metagenomic datasets also exist for the global ocean, such as the Tara Oceans dataset^15^. However, although the brackish Baltic Sea is composed of a mixture of marine- and freshwater like lineages^3,5,7,10^, these are genetically distinct from their relatives^8^, which hinders efficient read mapping to fresh- and marine water metagenomes. We here present a Baltic Sea metagenome co-assembly (BARM; BAltic sea Reference Metagenome) with annotated genes constructed from three sets of samples, selected to cover variation over geography, depth and season (Table 1 (available online only), Fig. 1; Data Citation 1).

After preprocessing of the reads, the 81 samples combined contained 586 billion bases in 2.6 billion read pairs. To allow the assembly of genes also from genomes having low abundance in individual samples, data from all samples were co-assembled. The resulting co-assembly consisted of 14 billion bases distributed over 22 million contigs. Out of these contigs, 2.4 million contigs were longer or equal to 1 kilobase. Functional and taxonomic annotation of genes is computationally demanding. For this reason, and since longer contigs were deemed to be more trustworthy, only genes found on the contigs >1 kilobase were subjected to functional and taxonomic annotations; 6.8 million genes were found on these.

**Figure 2:**
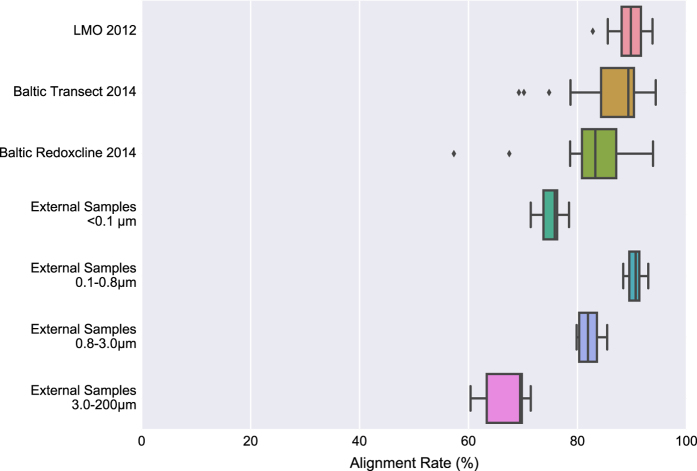
Mapping rates divided on different sample groups. Mapping rates are calculated by Bowtie2 (ref. 30) as the “overall alignment rate”. The three first sample groups; LMO 2012 (N=37, 0.2–3.0 μm), Baltic Transect 2014 (N=30, >0.2 μm) and Baltic Redoxcline 2014 (N=6, 0.2–3.0 μm; N=6, >3.0 μm; N=2, >0.2 μm) were included in the assembly, while the four last sample groups; External Samples <0.1 μm (N=6), External Samples 0.1–0.8 μm (N=6), External Samples 0.8–3.0 μm (N=6) and External Samples 3.0–200 μm (N=6) were not. The size intervals of the external samples indicate filter pore sizes used to tentatively separate viruses, free-living prokaryotes, and small and larger particles as well as Eukaryotic cells, respectively^34^. Created using Matplotlib^39^ and Seaborn^40^.

**Figure 3:**
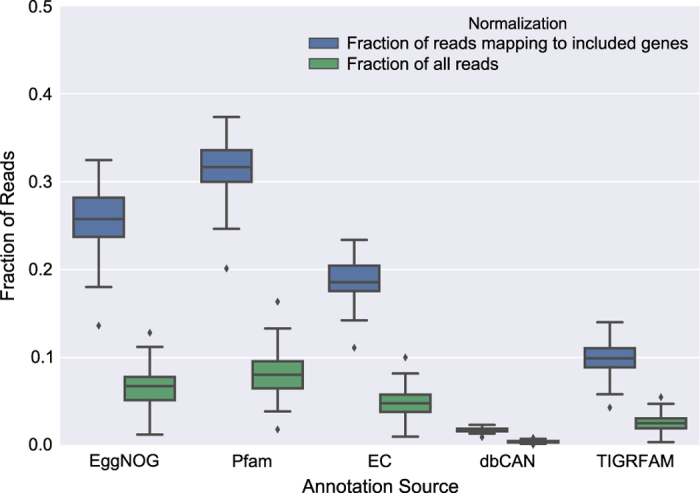
Fraction of reads mapping to genes annotated with respective database. Only genes identified on contigs longer than 1 kilobase were subjected to annotation, defining the ‘included genes’ category. N=81 for all categories. Created using Matplotlib^39^ and Seaborn^40^.

For functional analysis, several database sources were chosen; Pfam^16^, TIGRFAM (http://www.jcvi.org/cgi-bin/tigrfams/index.cgi), EggNOG^17^ and dbCAN^18^. Additionally, enzyme commission (EC) numbers^19^ were extracted based on the EggNOG assignments. Through mapping, the short reads were then used to quantify the individual genes over all the different samples, which were summarized per annotation identifier (ID) for each respective annotation source. The mapping rates for the different sample groups and annotation sources are summarized in Fig. 2 and Fig. 3, where in Fig. 2 also 24 samples from a published metagenomic study^20^ (Data Citation 2) of the Baltic Sea are included to illustrate the capabilities for BARM to work as a reference gene catalogue for the Baltic Sea.

Along with the dataset, a public web interface (BalticMicrobeDB) was constructed to facilitate exploratory analysis of the data (https://barm.scilifelab.se). Through this it is possible to view counts of functional and taxonomic annotations over the different sample groups. Moreover, it is possible to search for functional annotations based on their descriptive texts and choose to view or download the counts for only those matching the search query.

The annotated assembly presented here is a rich resource for further exploitation of the published datasets, facilitated through the web interface, but could also function as a reference metagenome assembly for the Baltic Sea, decreasing the computational demands for the analysis of new metagenome and metatranscriptome samples, and serve as reference for metaproteome analyses.

## Methods

### Sampling, DNA Extraction and Sequencing

The thirty seven surface-water (2 m depth) samples from the 2012 time series (March to December) from the Linneaus Microbial Observatory (LMO), station located 10 km off the east coast of Öland, and where the maximum depth is 47 m, have been described in Hugerth et al. (2015)^8^ (Data Citation 3). Briefly, after prefiltration through 3.0 μm, DNA was extracted from 0.2 μm Sterivex™ cartridge filters (Millipore) using the protocol described in Riemann et al. (2000)^21^ and sequenced on one HiSeq high-output flowcell with an average of 31.9 million pair-end reads per sample.

The 30 transect samples were taken during a cruise initiated by Leibniz Institute for Baltic Sea Research, Warnemünde on the R/V Alkor, carried out for the BONUS BLUEPRINT project from June 4 to June 17 2014. Samples for DNA analyses were collected using a compact CTD (profiling instrument that records conductivity, oxygen, temperature and depth) SBE 911 Plus with a SBE-rosette SBE32 (Sea Bird Electronics Inc., USA) equipped with 18×10 L FreeFlow-PWS-samplers (HYDRO-BIOS, Kiel, Germany). Water was sampled from oxic zones, in the range from 2 to 242 m depth, within the salinity gradient of the Baltic Sea. For DNA analysis, 1 L of seawater was directly filtered onto a 47 mm Durapore membrane filter with 0.2 μm pore size (GVWP04700, Merck Millipore, Darmstadt, Germany) by a vacuum of <300 mbar. Subsequently, the filters were folded, flash frozen using liquid nitrogen and stored at −80 °C until further processing. DNA was extracted using a modified protocol of the QIAamp DNA Mini Kit (51304, Qiagen, Hilden, Germany) with an initial bead-beating step and a cleanup and concentration process using the Zymo gDNA Clean and Concentrator Kit (D4010, Zymo Research Europe, Freiburg, Germany). The concentration and quality of the eluted DNA was assured by gel electrophoresis and Bioanalyzer DNA 12000 kit (5067-1508, Agilent Technologies, Santa Clara, USA). The samples were sequenced at the National Genomics Infrastructure at Science for Life Laboratory, Stockholm, Sweden, using a full HiSeq 2500 high-output flowcell producing an average of 69.5 million pair-end reads per sample.

The redoxcline samples consist of samples from station Boknis Eck (Data Citation 4), located at the entrance of the Eckernforde Bay in the southwestern Baltic Sea, and from station TF0271 at the Gotland Deep in the eastern Gotland Basin. The Boknis Eck station was sampled on September 23, 2014 on the R/V Littorina during routine monitoring activities performed monthly by the GEOMAR Helmholtz Centre for Ocean Research Kiel. Due to windy conditions before the sampling day, the water at the Boknis Eck station was mixed over most of the water column and only the bottom water was sulfidic. Water was sampled from the mixed oxygenated layer and from the sulfidic bottom water, which was captured on a 3 μm pore size membrane filters (Whatman, Maidstone, UK) followed by 0.2 μm pore size Sterivex-GV filters (Millipore Billerica, Massachusetts, USA). The Gotland Basin was sampled during the cruise EMB087 on the R/V Elisabeth Mann Borgese on October 18 and October 26, 2014. The samples from October 18 were taken in the context of an experiment close to the oxic-anoxic interface from suboxic and anoxic water layers and were captured directly on 0.2 μm pore size Durapore membrane filters (Whatman, Maidstone, UK). The samples from October 26 were taken to cover different zones in the redox gradient (suboxic, oxic-anoxic interface, upper sulfidic, lower sulfidic) and were captured first on a 3 μm pore size membrane filters (Whatman, Maidstone, UK) followed by 0.2 μm pore size Sterivex-GV filters. DNA from water captured on 3 μm pore size membrane filters and 0.2 μm Sterivex-GV filters was extracted using the QIAmp DNA Mini Kit (Qiagen, Hilden, Germany): ATL buffer was added to filter pieces together with 200 μm low-binding Zirconium beads (OPS Diagnostics, Lebanon, NY, USA) and the suspension was vortexed for 5 minutes at maximum speed. Subsequently proteinase K was added and the suspension was incubated for approximately 1h at 56 °C before continuing DNA extraction by following the manufacturer’s instructions. Nucleic acids from Gotland Basin water sampled on October 18 on 0.2 μm pore size membrane filters were extracted using the AllPrep DNA/RNA Mini Kit (Qiagen, Hilden, Germany). Similar as before, filters were vortexed together with Zirconium beads in RTL buffer before continuing nucleic acid extraction by following the manufacturer’s protocol. The concentration and quality of the eluted DNA was assured by gel electrophoresis. The samples were sequenced on a single HiSeq 2500 lane producing an average of 20.7 million pair-end reads per sample.

All sequencing libraries (including LMO) were prepared with the Rubicon ThruPlex kit (Rubicon Genomics, Ann Arbor, Michigan, USA) according to the instructions of the manufacturer.

### Preprocessing and Assembly

The quality of the reads were checked and visualized with FastQC (http://www.bioinformatics.babraham.ac.uk/projects/fastqc/) through MultiQC^22^ and trimmed from low quality bases with cutadapt^23^ using Phred score 15 as a cutoff. Adapter sequences were also removed using cutadapt, keeping only read pairs where both reads in the pair were longer than 31 bases. Preprocessed reads were then assembled using Megahit^24^ version 1.0.2 with default parameters including kmers 21,41,61,81 and 99.

Exclusively to the 30 samples from the transect cruise, genomic material (20 ng per L of seawater) from a known genome of *Thermus thermophilus* (strain HB8), which is not expected to be present in the Baltic Sea naturally, was added after filtration but prior to the DNA extraction, serving as internal standard to enable absolute quantifications. Aligning all contigs from the metagenome assembly against this reference genome showed that 84.1% of the genome was recovered within contigs aligning with average 99.82% identity. These additional genome contigs were kept in the reference assembly but reads aligning to the reference genome were filtered out before the quantification steps, and before uploading the processed reads to the European Nucleotide Archive (ENA) (Data Citation 5).

### Functional Annotation

Genes were predicted on all contigs using Prodigal^25^ version 2.6.3 with the ‘--meta’ tag which potentially uses different coding tables for different contigs. Genes located on contigs longer or equal to 1 kilobase, identified with the script toolbox/scripts/fasta_lengths.py, were used for functional and taxonomic annotation. For functional annotation, the databases EggNOG^17^, Pfam^16^, TIGRFAM (http://www.jcvi.org/cgi-bin/tigrfams/index.cgi) and dbCAN^18^ were chosen. Furthermore, EC-numbers^19^ were extracted from the EggNOG annotations.

To annotate genes with EggNOG^17^ IDs, the EggNOG hmm file for all organisms, NOG.hmm.tar.gz, version 4.5 was downloaded from http://eggnogdb.embl.de/download/eggnog_4.5/data/NOG/. For performance reasons, hmmsearch was used instead of hmmscan^26^, initially removing all hits with an E-value >0.0001. To select a maximum of one annotation per gene, the hit with highest score was chosen using the script toolbox/scripts/hmmer_filtering/keep_top_score.py. Information about each annotation was downloaded from http://eggnogdb.embl.de/download/eggnog_4.5/data/NOG/NOG.annotations.tsv.gz.

An Enzyme Commision (EC) number^19^ was assigned to each EggNOG through the Uniprot^27^ proteins included in the EggNOG model, if a majority of its EC-assigned members were assigned to that EC. Note that proteins could have multiple EC numbers assigned and therefore some EggNOGs were assigned multiple EC numbers. The files needed for the conversion were eggnog4.protein_id_conversion.tsv.gz (downloaded from http://eggnogdb.embl.de/download/eggnog_4.5/ on January 9th 2017) and NOG.members.tsv.gz (downloaded from http://eggnogdb.embl.de/download/eggnog_4.5/data/NOG/ on January 9th 2017). The protein ID conversion file gives EC numbers per reference protein and the members file gives the reference proteins that build each model. The protein with taxaid 400682 and protein ID “PAC” was removed from the protein ID conversion file since it had 695 EC entries. Likewise for taxaid 7070 and protein ID “TCOGS2”, with 686 EC entries. The protein ID with the third most entries had 6 entries and therefore the two others were deemed as outliers. The suspected reason is that these entries belong to different genes for these genomes but there were no way to resolve this and the EC-number assignment for each EggNOG was deemed to not be affected by this. Given the assignment of EC-numbers per EggNOG, the assignment per gene was done with toolbox/scripts/assign_ec_from_nog.py.

Annotation against the dbCAN^18^ (DataBase for automated Carbohydrate-active enzyme ANnotation) database was performed using version 5 (downloaded from http://csbl.bmb.uga.edu/dbCAN/download.php). Following the instructions from dbCAN (downloaded from http://csbl.bmb.uga.edu/dbCAN/download/readme.txt), hmmscan^26^ was used with the option --domtblout and the result was further treated with the recommended script hmmscan-parser.sh (reference of used script available within toolbox/third_party_scripts/dbcan/hmmscan-parser.sh) from dbCAN requiring a covered fraction of the HMM larger than 0.3 and keeping long alignments (>80 amino acids) if the E-value was less than 1e-5 and short alignments if the E-value was less than 1e-3. An additional script toolbox/hmmer_filtering/dbcan_strict_filtering.py was applied, choosing to follow recommendations for bacteria from dbCAN, keeping annotations with e-value less than 1e-18 and alignment coverage greater than 0.35. To allow for more than a single domain within a gene, any annotation which fulfilled these criteria was kept. Information about each annotation was collected (downloaded from http://csbl.bmb.uga.edu/dbCAN/download/FamInfo.txt).

Annotation against Pfam^16^ version 30.0 was conducted with the script pfam_scan.pl supplied from the ftp://ftp.ebi.ac.uk/pub/databases/Pfam/Tools for version 28.0, using hmmer version 3.1b1 (ref. 26). To allow for more than a single domain within a gene, any annotation which fulfilled these criteria was kept. Information about each annotation was collected as columns 1,2 and 4 from the file pfamA.txt.gz downloaded from ftp://ftp.ebi.ac.uk/pub/databases/Pfam/releases/Pfam30.0/database_files/ on January 11th 2017.

Annotation against TIGRFAM version 15, was performed using hmmsearch (v. 3.1b2)^26^ with --cut_tc argument to filter models by trusted cutoff. For each protein sequence, the best scoring HMM was selected using hmmparse.py available at https://github.com/johnne/biotools/blob/master/scripts/hmmparse.py

### Taxonomic annotation

The method used to assign taxonomy was chosen in order to assign as many contigs as possible to a taxonomy while still keeping false positives to a low level. As the number of sequences in reference databases closely related to the genomes in our samples was expected to be low^8^, amino acid sequences from the assembly were used to compare against other amino acid sequences in the reference database, enabling higher sensitivity (due to the more conserved nature of amino acid sequences). This comparison was done using Diamond version 0.8.26 (ref. 28) with the parameters “--seg yes”,”--sensitive” and “--top 10” against the NCBI nr database downloaded December 2nd 2016.

The code used to assign taxonomy from the Diamond search was based on an original available in the DESMAN package^29^ and the modified version of the code is available as the script toolbox/scripts/taxonomy_from_genes_to_contigs/lca_per_contig.py. The assignment was done as follows: all reported hits from the Diamond search were given a weight based on the aligned fraction of the query and the percentage identity of the alignment. At each taxonomic level, if the sum of the weights for one taxon was greater than half the sum of all weights, the gene was assigned to that taxon as long as the percentage identity was high enough. The levels for the percentage identity were set to 40% at superkingdom level, 50% at phylum level, 60% at class level, 70% at order level, 80% at family level, 90% at genus level and 95% at species level.

Taxonomic assignments were set per contig to the most detailed level where consensus for at least 50% of the weights of the preliminary gene assignments could be achieved. Genes without taxonomic annotation were ignored. The shared assignment was propagated to all genes present on that contig. In this way, all genes present on one contig will always share the taxonomic assignment. If no single superkingdom accounted for a majority of the gene assignment weights for a contig, the contig was left unassigned.

### Quantification and Normalization

To use the metagenome assembly as a reference assembly, individual samples are functionally and taxonomically annotated by quantifying the different annotations present in the assembly. This is done by mapping all short reads against the assembly and quantifying genes, and thereby any associated annotation, with the number of reads mapping to them. More specifically bowtie2 (ref. 30) version 2.2.6 was used with the parameter “--local” for mapping, duplicated reads were removed with picard version 1.118, bam-file sorting was done with Samtools^31^ version 1.3, and the htseq-count script from htseq^32^ version 0.6.1 was used to get raw counts per gene. Counts per annotation was achieved by summing all counts for genes annotated with each respective annotation.

When quantifying annotation types where multiple annotations were allowed for a single gene (dbCAN and Pfam), some genes contributed several times to the quantities. This was kept in order to facilitate analysis of differential abundance for the individual annotations.

Along with raw counts of reads for each annotation type and taxonomy, a count normalized by gene length and number of mapped reads was also calculated. Analogously to the formula for Transcripts Per Million used in transcriptomics (ref 33), we calculate TPM for gene counts:

$$TPM=\frac{r_{g}\cdot rl\cdot 10^{6}}{fl_{g}\cdot T}$$

$$T=\sum_{g\in G}\frac{r_{g}\cdot rl}{fl_{g}}$$

Where *r*_*g*_ is the number of reads mapped to gene *g* from the sample, *rl* is the average read length for the sample, *fl*_*g*_is the length of the gene and *G* is the set of all genes. T is a convenience variable for the indicated sum over all genes.

### Code availability

Code used to preprocess reads, assemble contigs and annotate genes is publicly available at https://github.com/EnvGen/BLUEPRINT_pipeline, containing the pipeline definition of the workflows used, https://github.com/EnvGen/snakemake-workflows, where the snakemake rules are specified in order to build the command used for each step, and the branch BARM_publication of https://github.com/EnvGen/toolbox, for custom scripts. Scripts within the latter repository that have been used have been indicated throughout the text.

## Data Records

The preprocessed sequencing reads from the Transect and Redoxcline samples were submitted to ENA hosted by EMBL-EBI under the study accession number PRJEB22997 (Data Citation 5). The raw reads from LMO were published elsewhere^8^ and are accessible at NCBI (Data Citation 3). Contig, gene and protein sequences from the co-assembly of the Transect, Redoxcline and LMO samples, as well as quantification tables, contextual data for the samples, and the annotations for each gene are accessible on Figshare (Data Citation 1). The raw sequencing reads from the external samples used for evaluation were also published elsewhere^34^ and are accessible at NCBI (Data Citation 2).

## Technical Validation

The mapping rates for all samples included in the reference assembly are shown in Fig. 2, where the majority of samples included in the assembly reaches a level above 80%. This serves as a validation of the completeness of the metagenome assembly. The fraction of reads that did not map to the coassembly, and were hence not assembled past the 200 bases length cutoff most likely originate from low abundance species, or species with high intraspecies diversity generating fragmented assemblies. The mapping rate of the external samples shows the capability for this assembly to serve as a reference metagenome assembly for the Baltic Sea. These external samples^34^ were collected in a different year (2011) and a station (58.82 N 17.63 E) separate from where the samples included in the assembly were taken. This represents a realistic scenario where BARM is used as a reference metagenome for the Baltic Sea. The mapping rates vary with the filter fractions, where reads originating from the largest (3.0–200 μm) and smallest (<0.1 μm) fractions displayed lower rates than the two intermediate fractions (0.1–0.8 μm and 0.8–3.0 μm), indicating that picoplankton are better represented in BARM than larger eukaryotic plankton and viruses.

Assignment rates for different annotation types, as shown in Fig. 3, are in the majority of cases below 10% of the total number of reads, which is expected since only genes on long contigs (representing 40% of the bases of the total assembly) were predicted and subjected to annotation. The fraction of reads annotated among reads mapping to genes included in the annotation procedure reaches well over 30% for Pfam and shows the generality of that database as compared to i.e. dbCAN, a much more niched resource, which reaches only around 2% of reads mapping to genes included in the annotation.

**Figure 4:**
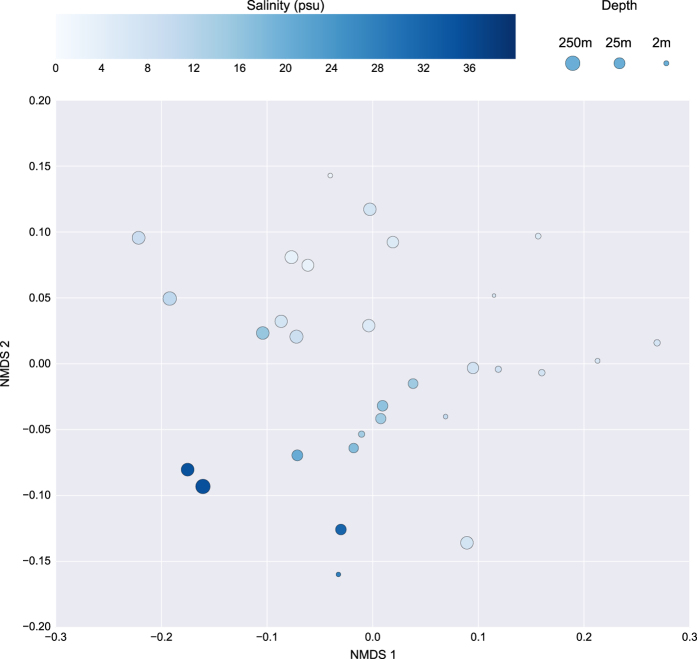
Non-metric dimensional scaling (NMDS) of the 30 samples included in the Transect sample group based on EggNOG annotation. Samples are colored and sized according to salinity and depth, respectively. Created using Matplotlib^39^ and Seaborn^40^.

The functional annotation was further validated through an NMDS plot (Fig. 4) based on the EggNOG annotations of the transect data. Depth was found to be negatively correlated with the first dimension (Spearman’s rank correlation *ρ*=−0.73, *P*=5.4^−06^) and salinity was negatively correlated with the second dimension (Spearman’s rank correlation *ρ*=−0.77, *P*=2.4^−06^). These two environmental parameters have previously been found to correlate strongly with the microbial community in the Baltic Sea^5^ which strengthens our trust in the EggNOG annotations. Furthermore, analyzing a single annotation with a known function, namely the photosynthetic reaction centre protein (PF00124), we could see a strong negative correlation with sampling depth over the thirty transect samples (Spearman correlation coefficient ρ=−0.87, *P*=3.1^-10^).

**Figure 5:**
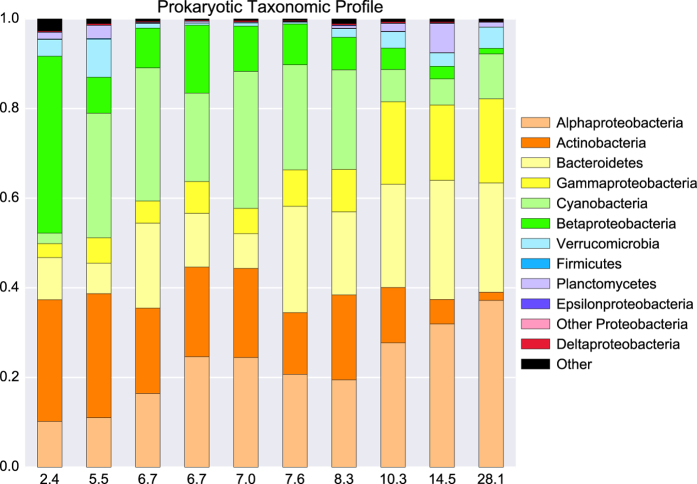
Taxonomic profiles of the 10 transect samples obtained from surface waters. Numbers on x-axis indicate salinity, given in practical salinity units (PSU), and are sorted with increasing salinity to the right. Created using Matplotlib^39^ and Seaborn^40^.

The taxonomic annotation was validated by inspecting the taxonomic profile of the transect samples. The same dominant prokaryotic taxonomic groups were observed as in previous pan-Baltic amplicon sequencing and metagenomic studies^5,7,10,11^, and the overall trends were conserved with an increase in *Alpha-* and *Gammaproteobacteria* and a decrease in *Actinobacteria* and *Betaproteobacteria* with increasing salinity levels (Fig. 5).

Among the predicted proteins in BARM, 98% lacked hits with amino acid identities above 95%, hence potentially representing species for which sequenced genomes are lacking^35^. 31% of the sequences lacked significant hits (E-value >1) and potentially correspond to novel protein families.

## Usage Notes

A publicly available repository at https://github.com/EnvGen/BARM_tools hosts instructions and a pipeline on how to quantify genes and their annotations within BARM for any kind of Baltic Sea metagenomic and metatranscriptomic samples.

The web interface BalticMicrobeDB, available to the public at http://barm.scilifelab.se, can be used to explore and access data for the three sample sets that the assembly is based upon. At the index page, the user can choose whether to access functional annotations or taxonomic annotations. For the functional annotations, the user can select specific annotation sources and identifiers and select the sample groups for which the counts will be displayed. Furthermore, a text search over the identifiers and the descriptions of the annotations can be used to create a custom table of counts over the selected samples. For taxonomic annotations, counts for the top level superkingdom are first presented but the user can unfold a taxonomic tree to select any taxon to view counts for.

## Additional information

**How to cite this article**: Alneberg, J. *et al.* BARM and BalticMicrobeDB, a reference metagenome and interface to meta-omic data for the Baltic Sea. *Sci. Data* 5:180146 doi: 10.1084/sdata.2018.146 (2018).

**Publisher’s note**: Springer Nature remains neutral with regard to jurisdictional claims in published maps and institutional affiliations.

## Supplementary Material



## Figures and Tables

**Table 1:** Sample summary.

Sample Group	Sample	Station	Sampling Depth (m)	Sampling Date	Filter-size (μm)	Prefiltration size (μm)	Latitude	Longitude	Salinity (psu)	Oxygen (μmol/kg)
Lmo	120314	LMO	2.0	2012-03-14	0.2	3.0	56.9384	17.062	6.7	not collected
Lmo	120322	LMO	2.0	2012-03-22	0.2	3.0	56.9384	17.062	6.9	not collected
Lmo	120328	LMO	2.0	2012-03-28	0.2	3.0	56.9384	17.062	6.5	not collected
Lmo	120403	LMO	2.0	2012-04-03	0.2	3.0	56.9384	17.062	6.5	not collected
Lmo	120416	LMO	2.0	2012-04-16	0.2	3.0	56.9384	17.062	6.7	not collected
Lmo	120419	LMO	2.0	2012-04-19	0.2	3.0	56.9384	17.062	6.7	not collected
Lmo	120423	LMO	2.0	2012-04-23	0.2	3.0	56.9384	17.062	6.7	not collected
Lmo	120507	LMO	2.0	2012-05-07	0.2	3.0	56.9384	17.062	6.6	not collected
Lmo	120516	LMO	2.0	2012-05-16	0.2	3.0	56.9384	17.062	6.4	not collected
Lmo	120521	LMO	2.0	2012-05-21	0.2	3.0	56.9384	17.062	6.5	not collected
Lmo	120531	LMO	2.0	2012-05-31	0.2	3.0	56.9384	17.062	6.5	not collected
Lmo	120604	LMO	2.0	2012-06-04	0.2	3.0	56.9384	17.062	6.5	not collected
Lmo	120613	LMO	2.0	2012-06-13	0.2	3.0	56.9384	17.062	6.5	not collected
Lmo	120619	LMO	2.0	2012-06-19	0.2	3.0	56.9384	17.062	6.5	not collected
Lmo	120628	LMO	2.0	2012-06-28	0.2	3.0	56.9384	17.062	6.4	not collected
Lmo	120705	LMO	2.0	2012-07-05	0.2	3.0	56.9384	17.062	6.5	not collected
Lmo	120709	LMO	2.0	2012-07-09	0.2	3.0	56.9384	17.062	6.5	not collected
Lmo	120717	LMO	2.0	2012-07-17	0.2	3.0	56.9384	17.062	6.2	not collected
Lmo	120802	LMO	2.0	2012-08-02	0.2	3.0	56.9384	17.062	6.2	not collected
Lmo	120806	LMO	2.0	2012-08-06	0.2	3.0	56.9384	17.062	6.2	not collected
Lmo	120813	LMO	2.0	2012-08-13	0.2	3.0	56.9384	17.062	6.2	not collected
Lmo	120820	LMO	2.0	2012-08-20	0.2	3.0	56.9384	17.062	6.2	not collected
Lmo	120823	LMO	2.0	2012-08-23	0.2	3.0	56.9384	17.062	6.2	not collected
Lmo	120828	LMO	2.0	2012-08-28	0.2	3.0	56.9384	17.062	6.1	not collected
Lmo	120903	LMO	2.0	2012-09-03	0.2	3.0	56.9384	17.062	6.2	not collected
Lmo	120910	LMO	2.0	2012-09-10	0.2	3.0	56.9384	17.062	6.1	not collected
Lmo	120920	LMO	2.0	2012-09-20	0.2	3.0	56.9384	17.062	6.4	not collected
Lmo	120924	LMO	2.0	2012-09-24	0.2	3.0	56.9384	17.062	6.2	not collected
Lmo	121001	LMO	2.0	2012-10-01	0.2	3.0	56.9384	17.062	6.4	not collected
Lmo	121004	LMO	2.0	2012-10-04	0.2	3.0	56.9384	17.062	6.3	not collected
Lmo	121015	LMO	2.0	2012-10-15	0.2	3.0	56.9384	17.062	6.2	not collected
Lmo	121022	LMO	2.0	2012-10-22	0.2	3.0	56.9384	17.062	6.2	not collected
Lmo	121028	LMO	2.0	2012-10-28	0.2	3.0	56.9384	17.062	6.2	not collected
Lmo	121105	LMO	2.0	2012-11-05	0.2	3.0	56.9384	17.062	6.2	not collected
Lmo	121123	LMO	2.0	2012-11-23	0.2	3.0	56.9384	17.062	6.5	not collected
Lmo	121128	LMO	2.0	2012-11-28	0.2	3.0	56.9384	17.062	6.4	not collected
Lmo	121220	LMO	2.0	2012-12-20	0.2	3.0	56.9384	17.062	6.2	not collected
Redox	P2236_101	TF0271	110.0	2014-10-26	3.0	-	57.3222	20.0506	10.9796	23.96521144
Redox	P2236_102	TF0271	110.0	2014-10-26	0.2	3.0	57.3222	20.0506	10.9796	23.96521144
Redox	P2236_103	TF0271	120.0	2014-10-26	3.0	-	57.3222	20.0506	11.282	5.9394045
Redox	P2236_104	TF0271	120.0	2014-10-26	0.2	3.0	57.3222	20.0506	11.282	5.9394045
Redox	P2236_105	TF0271	140.0	2014-10-26	3.0	-	57.3222	20.0506	11.7535	0.0
Redox	P2236_106	TF0271	140.0	2014-10-26	0.2	3.0	57.3222	20.0506	11.7535	0.0
Redox	P2236_107	TF0271	200.0	2014-10-26	3.0	-	57.3222	20.0506	12.1052	0.0
Redox	P2236_108	TF0271	200.0	2014-10-26	0.2	3.0	57.3222	20.0506	12.1052	0.0
Redox	P2236_109	Boknis Eck	10.0	2014-09-23	3.0	-	54.5381	10.0478	16.276	276
Redox	P2236_110	Boknis Eck	10.0	2014-09-23	0.2	3.0	54.5381	10.0478	16.276	276
Redox	P2236_111	Boknis Eck	25.0	2014-09-23	3.0	-	54.5381	10.0478	25.082	0
Redox	P2236_112	Boknis Eck	25.0	2014-09-23	0.2	3.0	54.5381	10.0478	25.082	0
Redox	P2236_113	TF0271	139.0	2014-10-18	0.2	-	57.3228	20.0606	11.7173	0
Redox	P2236_114	TF0271	100.0	2014-10-18	0.2	-	57.3228	20.0606	10.5491	41.53
Transect	P1994_116	MO7	2.8	2014-06-04	0.2	-	54.6979	12.7047	8.325	333.16
Transect	P1994_117	MO7	12.7	2014-06-04	0.2	-	54.6979	12.7047	14.969	268.41
Transect	P1994_118	MO7	19.3	2014-06-04	0.2	-	54.6979	12.7047	16.415	240.72
Transect	P1994_101	AT1	1.8	2014-06-05	0.2	-	58.1323	10	28.05	288.5
Transect	P1994_102	AT1	80.1	2014-06-05	0.2	-	58.1323	10	34.914	281.36
Transect	P1994_103	AT1	241.7	2014-06-05	0.2	-	58.1323	10	35.081	273.77
Transect	P1994_110	MO3	2.9	2014-06-06	0.2	-	56.867	11.4339	14.526	303.69
Transect	P1994_111	MO3	11.4	2014-06-06	0.2	-	56.867	11.4339	17.613	308.15
Transect	P1994_112	MO3	20.8	2014-06-06	0.2	-	56.867	11.4339	31.943	190.7
Transect	P1994_113	MO6	1.7	2014-06-07	0.2	-	54.2821	11.568	10.28	316.64
Transect	P1994_114	MO6	16.3	2014-06-07	0.2	-	54.2821	11.568	14.676	283.14
Transect	P1994_115	MO6	22.6	2014-06-07	0.2	-	54.2821	11.568	20.323	194.72
Transect	P1994_119	S6	3.0	2014-06-08	0.2	-	55.5659	16.3665	7.577	342.54
Transect	P1994_120	S6	30.3	2014-06-08	0.2	-	55.5659	16.3665	7.661	339.86
Transect	P1994_121	S6	66.6	2014-06-08	0.2	-	55.5659	16.3665	15.73	16.52
Transect	P1994_104	AT3	1.4	2014-06-09	0.2	-	57.3057	20.0766	6.744	380.95
Transect	P1994_105	AT3	65.4	2014-06-09	0.2	-	57.3057	20.0766	7.226	317.98
Transect	P1994_106	AT3	116.5	2014-06-09	0.2	-	57.3057	20.0766	10.862	9.38
Transect	P1994_125	S10	2.4	2014-06-10	0.2	-	61.7829	19.2952	5.456	391.22
Transect	P1994_126	S10	33.9	2014-06-10	0.2	-	61.7829	19.2952	5.462	393.45
Transect	P1994_127	S10	55.8	2014-06-10	0.2	-	61.7829	19.2952	5.566	364.87
Transect	P1994_107	AT4	1.8	2014-06-12	0.2	-	65.446	23.2973	2.442	389.88
Transect	P1994_108	AT4	42.3	2014-06-12	0.2	-	65.446	23.2973	3.04	414.89
Transect	P1994_109	AT4	78.7	2014-06-12	0.2	-	65.446	23.2973	3.146	401.05
Transect	P1994_122	S7	1.9	2014-06-16	0.2	-	58.5849	18.2327	6.742	345.22
Transect	P1994_123	S7	60.5	2014-06-16	0.2	-	58.5849	18.2327	7.248	338.08
Transect	P1994_124	S7	76.5	2014-06-16	0.2	-	58.5849	18.2327	9.221	17.42
Transect	P1994_128	TF245	3.0	2014-06-17	0.2	-	57.1162	17.6642	7.05	339.42
Transect	P1994_129	TF245	55.9	2014-06-17	0.2	-	57.1162	17.6642	7.304	343.44
Transect	P1994_130	TF245	85.7	2014-06-17	0.2	-	57.1162	17.6642	8.902	27.69
Important contextual parameters for the samples, which were used to construct the co-assembly.										

## References

[d1] figshare Alneberg J. Andersson A. F 2018 https://doi.org/10.6084/m9.figshare.c.3831631

[d2] NCBI Sequence Read Archive 2016 SRP077551

[d3] NCBI Sequence Read Archive 2015 SRP058493

[d4] PANGAEA Bange H. W. Malien F. 2015 https://doi.org/10.1594/PANGAEA.855693

[d5] European Nucleotide Archive 2017 ERP104730

